# It is time to review how unlicensed medicines are used

**DOI:** 10.1007/s00228-015-1886-z

**Published:** 2015-07-09

**Authors:** Adam Sutherland, Stephen Waldek

**Affiliations:** Royal Manchester Children’s Hospital, Oxford Road, Manchester, M13 9WL UK; 31 Harboro Road, Manchester, M33 5AN UK

**Keywords:** Pharmaceutical preparations, Unlicensed medicines, Patient safety, Medicine regulation, Adverse drug reactions, Professional liability

## Abstract

The safe and effective use of medicines is an integral part of the medicine safety agenda. We present a phenomenological topic review of the literature relating to the use of unlicensed medicines (ULM). There is evidence to demonstrate that the use of ULM is associated with increased incidence of adverse drug reactions, and that despite advances in medicine regulation and guidance from professional organisations, the use of ULM in at risk populations has not reduced. There is also evidence to suggest that patients and their carers are not being provided with adequate information about their medicines and that ULM are being used where safer licensed alternatives are available. This is contrary to the philosophy of “patient-focussed care”. We conclude that organisational governance processes and professional guidelines have not kept pace with regulatory developments or changes in legal and ethical understanding. We recommend that governance procedures for ULM be updated across healthcare settings to ensure that patients are involved in the decisions made about their medicines including the regulatory status of the medicine. This includes ensuring adequate consent is obtained from the patient (or their advocate). We also recommend that professional bodies clarify their position on when ULM can be used instead of licensed medicines to ensure that licensed medicines are used wherever possible. In the current economic environment, commissioners and clinicians must resist the temptation to use lower-quality ULM in place of licensed ones to cut costs. We go on to recommend areas of further research including the extent of ULM prescribing where licensed alternatives exist and the geographical and social factors that influence clinician prescribing of ULM.

## Introduction

The correct use of medicines constitutes an important part of the quality of care and patient safety agenda. All healthcare professionals and the organisations for which they work, need to ensure that the procurement, prescribing, dispensing and administration of medicines complies with best practice so helping to ensure, effective, safe, and patient focused use of all medicines. Within the medicines management quality and safety agenda, one issue that needs to be addressed is the widespread use of unlicensed medicines (ULM) in its various guises. We have reviewed the evidence around the use of ULM and suggest how arrangements, at all levels, might be adapted or enforced to ensure that risks to patients from this practice are mitigated, while still considering individual patients’ needs.

### Definitions

There are three situations covered by the term ULM, and we consider these as separate entities.

1. “Off label” (OL) refers to a situation where a licensed medication is used outside the terms of its licensed indication(s). This also refers to use in doses or routes of administration not in the authorisation, as well as to use outside the stipulated age ranges and administration to patients with a cited contraindication [1, 2].
2. A “specials medicine” (SM) is an unlicensed medicinal product prepared by a licensed specials manufacturer of an unlicensed product to meet the specific needs of a patient at the request of an authorised healthcare professional [3]. There is a provision in the regulations to permit these manufacturers to produce batches of these medicines providing there is data to support and inform the physicochemical stability of these medicines.
3. The literature considers SM and true unlicensed medicines (TULM) together which can be confusing. A TULM is defined as a medicine that is used without marketing authorisation in the country of use [3]. There are different issues around prescribing, supply, and administrating of these medicines. Therefore we will consider SM and TULM separately.

Pharmacies are required to maintain records of the dispensing of SM and TULM to ensure accountability [4]. However, prescribers have no such obligation. While TULM use is uncommon, data is sparse because in many cases use only comes to light when an adverse incident occurs [5].

## Methods

We reviewed published literature via PubMed, MedLine and EmBase using the following key words—unlicensed medicines, medicines regulation, medicines safety, off-label prescribing, and specials medicines. In addition, we also hand-searched references for relevant additional citations. We also reviewed regulatory documents produced by the Medicines and Healthcare Products Regulatory Agency (MHRA) and European Medicine Agency (EMA) as well as guidance from professional bodies. An internet search using the terms “unlicensed medicines” and “unregistered medicines” was undertaken. The references and other documents we obtained were then used to undertake and phenomenological review of the topic.

## Results

There is substantial evidence of ULM use, mostly within paediatric populations. The first empirical study was in 1996 [6] with several subsequent publications [1, 7–10]. In 2004, the EMA published “Evidence of harm from off-label or unlicensed medicines in children” [11]. This regulatory review found, as for adults, that there was under-reporting of adverse drug reactions (ADRs) and that this was less so with prospective data collection. Clinically apparent ADRs were more common with ULM compared to licensed medicines. The incidence of ADRs doubled when both clinical and laboratory parameters of detection were used [11]. A prospective review using “EudraVigilance” reports (an electronic data processing network and management system for reporting and evaluating drug reactions [11]) demonstrated there were 820 serious ADRs involving ULM in children between 2001 and 2002 including 361 patients who either needed hospitalisation or where hospitalisation was lengthened; 103 children died [11]. This report implies that the use of OL and TULM is associated with no proper labelling (side effects, cautions, and contraindications) and dosing instructions, making medication errors more likely. An earlier study from the UK reported a significant association between ULM and ADRs in children (RR 1.27, 95 % CI 1.21–1.34) [12]. Looking at individual reports in the paediatric population, there is some variability in the percentage of prescriptions for ULM. A UK study reported that 36 % of prescriptions were for ULM with the majority being for OL use rather than TULM [1]. However, a Finnish study over 3 weeks in three paediatric wards reported 76 % of prescriptions contained at least one ULM (66 % OL, 33 % TULM) [13], The inclusion of the neonatal intensive care unit (NICU) contributes substantially to this result and is confirmed by Jain and colleagues who found 50 % of prescriptions in the NICU were for ULM [14]. Figures from community-based studies reveal similar results. A Dutch cross-sectional study found 22.7 % of prescriptions were for ULM [15] and 28.9 % were ULM in a population-based cohort study [16]. A Canadian primary care study in 2012 found a prevalence of ULM prescribing of 11 %. Of significance in this study was that careful review of the cases revealed that 79 % of them lacked good evidence of efficacy [17]. Psychotropic and other central nervous system preparations are common and are only included because of a patient’s age, changes to dose, route of administration, formulation, or indication that does not appear in the SPC. Only one report gives details of TULM with the two most common reasons given being a special formulation (SM) or modification of a licensed medicine (e.g. crushing or dispersing tablets or opening capsules). Examples of TULM identified by this study include a new medicine under a special manufacturing licence, a chemical used as a medicine, a medicine made from raw materials, and an imported medicine licensed in another country but not in the country of study [2].

Very little data is available for adults, but a recent report of a prospective, longitudinal study from Spain in five public hospitals over a 12-month period found 232 requests for ULM [18]. Unlike children, the top two medicine groups prescribed were monoclonal antibodies (rituximab 21.1 %) and muscle relaxants (botulinum toxin 10.7 %). Further analysis showed that in only 48.2 % the supporting evidence was high (levels 1 and 2). Significant side effects were present in 25.7 % of cases, but these were mostly predictable.

In paediatric and adult populations, we see that the level of supporting evidence for the use of ULM is low [19, 20] and this needs to be explored. O’Connor and Liddle, reporting on data collected on the OL use of rituximab, state that “the evidence base for this remains a challenge” [19]. In the field of oncology, things may be slightly better and could indicate a method of dealing with the ULM problems. Mellor and colleagues reviewed all the chemotherapy protocols in their Australian oncology centre and found that out of 448 protocols, 189 (42.2 %) advocated the use of ULM, and of these, 139 (69.9 %) had a good evidence base, while a further 39 (20.6 %) were based on phase II or III clinical trial data [20].

The formulation of a medicine is extremely important. Standing and colleagues reviewed paediatric trials and found that in many cases, information on formulation was lacking making the data difficult to validate [21]. They go on to point out that splitting, cutting, and dismantling tablets or capsules can have a significant impact on efficacy. They also state that different formulations can give rise to very different pharmacokinetics. Although this study was confined to paediatrics, the same would apply to all age groups. While OL use does not usually involve alteration of the medicine, the use of SM and TULM often requires reformulation or compounding of a product where there is no evidence of bioequivalence on which to base dose. Where a TULM is produced to be used instead of a licensed one containing the same substance, there is no guarantee that the unlicensed product has the same pharmacokinetic properties or therapeutic effect.

The literature presents problems that need to be addressed, and there are also issues that are evident but not explicitly included in the literature. While it is clear that the use of ULM is widespread, both in paediatric and adult prescribing practice, there is little written about the governance issues that surround this practice. The General Medical Council (GMC), in its “Good Practice Guidelines” (2013), gives clear guidelines on the prescribing of ULM [22]. While stating the circumstances under which ULM can be used, it is clear that prescribers should not use an unlicensed medicine where an identical licensed one is available. They also state that the prescriber must satisfy themselves on the safety and efficacy of the medicine they choose and that a good evidence base exists for their actions. The patient and their parent, guardian, or carer must be given information as to why an ULM is being prescribed and on what evidence. If the use of the medicine outside the licence is routine—such as the use of antibiotics in an excluded age group—then detailed information may not be necessary, and a brief explanation may suffice. Associated with the information, the prescriber should make adequate records as to why the ULM is being used and that information has been given to the patient or their parents. The whole issue of consent when dealing with ULM is covered by GMC guidance from 2008 [23]. Similar advice is also given by the MHRA in an article written in 2009 [24]. Both documents point out that the prescriber is responsible for all the consequences of using ULM. However, responsibility also rests with the dispenser. This means that there needs to be good communication between the two professional groups. In hospitals, this might happen through formulary committees and pharmacists’ involvement in the multidisciplinary team. Within the community, it is the responsibility of the dispensing pharmacist to contact the prescriber and discuss any ULM use. The roles and responsibilities of the nurse are also recognised [25]. All relevant regulators state that there needs to be careful monitoring for ADRs in all patients receiving ULM [11, 20, 22], and that in many cases, such as in oncology, the surveillance should be long term because of the possibility of very late complications [26].

## Discussion

As we have demonstrated, adverse events are under reported and this is almost certainly greater in the case of ULM use. Therefore, pharmacovigilance becomes a serious issue and the EMA and the MHRA emphasise this point. A landmark study from the UK demonstrated that OL and TULM use was associated with a significantly higher risk of an ADR in children (relative risk 1.67, with a 25 % increase in RR for every additional ULM prescribed) [27]. Companies marketing licensed products within the European Union (EU) are obliged to have pharmacovigilance systems in place and bear liability for the effects of their products used by patients under the terms of the marketing authorisation [28]. While prescribers and dispensers should be obliged to report all adverse events to the appropriate authorities (using the “yellow card” scheme in the UK), with SM and TULM, there is no such obligation for manufacturers to provide pharmacovigilance because all product liability rests with the prescriber and the multidisciplinary team (MDT). Prescribers and dispensers, as well as their organisations, should ensure that they have adequate liability insurance to cover ADRs from ULM.

Prescribers are increasingly being asked to prescribe using generic names for products, and there must be systems in place to ensure that the product dispensed is one that is licensed and not a cheaper unlicensed version. For example, in the EU, betaine (indicated for the treatment of homocystinuria), is available as a licensed product (Cystadane®, Orphan Europe) but in the UK, it is possible to source a cheaper unlicensed product as a SM. A physician prescribing betaine generically will need assurance that the licensed version is being dispensed.

### Ethical and legal considerations

The European ethical and legal situation has been well reviewed by Lenk and Duttge in 2014 [32]. They raised very clear issues about communication with patients and postulate that physicians and patients’ information needs are diametrically opposed. Patients consider the licensed status of their medicines as important when making choices, whereas physicians do not think patients need to take that into account. Among parents of children in a German renal clinic, only 24–30 % of parents knew the licensed status of their medicines and identified a small risk that parents would refuse unlicensed medicines or only use them if there was no other alternative available [32]. Within a palliative care setting, only 15 % of institutions regularly informed patients that they were receiving ULM and 22 % of prescribers stated that they never draw attention to the use of an ULM [33]. Quoting the work of Martin-Latry and colleagues on the ULM use of psychotropic medicines [34], Lenke and colleagues were not only worried about the extent of OL prescribing—97 % for anticonvulsants—but that many of the patients could be described as “vulnerable” yet there was no clarity around patient knowledge [35]. Responsibility for safety must consider the use of ULM as a safety issue and take therapeutic decisions in partnership with patients.

Case law and local regulations are out of the remit of this paper as these vary from country to country. However, Peter Feldschreiber of 4 New Square Chambers, London, has written on the topic of ULM from a UK perspective [36].

The MHRA issued a guidance note on the prescribing of caffeine, used in the treatment of apnoea of premature infants, to ensure that licensed products and doses were used [29]. The MHRA moved to restrict importation of unlicensed melatonin products after a licensed product became availabe. Melatonin is used for sleep disorders in many conditions and several unlicensed medications were available prior to a medicine being licensed [30]. To avoid restricting access to products for individual patients, the MHRA provided guidance and governance arrangements for unlicensed formulations that could be used.

### Patient considerations

There is a need for medicines to meet specific patient needs where there are no licensed alternatives. In the UK, companies exist that will provide SM that are often reformulations of licensed medicines. These are often manufactured in batches with the pharmacy dispensing them as needed on a named patient basis. This is commonly used for cardiovascular and neurological medicines as well as those used for rare diseases in children (e.g. captopril for heart failure and sodium phenylbutyrate in the management of raised ammonia levels in urea cycle disorders). This is legitimate activity but both prescriber and dispenser need to be aware of the potential risks and ensure that the drugs are prepared by a licensed manufacturer and that they have appropriate arrangements should there be an ADR. However, there are situations where care needs to be taken. Some licensed special manufacturers (including NHS hospitals) manufacture their own medicines—some of them identical to licensed products—and sell these on to other hospitals as TULM. This can lead to great variability in active medicine as was demonstrated in the case of 3, 4-diaminopyridine used for the treatment of Eaton-Lambert Myaesthenic syndrome. A marketing authorisation for this (as amifampridine phosphate, Firdapse®) was granted by the EMA in 2014. Prior to this, a number of licensed specials companies manufactured 3,4-diaminopyridine capsules from raw chemical. Green and colleagues analysed samples of these for medicine content and found large variations [31]. No sample achieved Good Manufacturing Practice standards of 95–105 % declared label content with some samples only reaching 22.5 % of declared content and some measuring 125 % of declared content.

The same issues are faced when a pharmacist or nurse takes a licensed medicine and reformulates it to make it more palatable for a patient. Even more interestingly, what of the nurse who crushes a tablet or opens a capsule so as to facilitate a patient swallowing the medicine, but thereby alters the efficacy or other attributes of the medicine making an ADR or poor response more likely? When these situations occur, there needs to be discussion between the pharmacist and the prescriber and administering nurse, as well as with the patient. This is not just a problem with young children, it also occurs with the elderly and with those with swallowing difficulties. Multi-disciplinary team (MDT) education is essential.

### Decision-making considerations

Increasingly, medicines are being used OL because innovative indications are being identified with clear patient benefit. Innovation is crucial to medical progress and, provided there is good evidence for this approach, patients should not be denied what some might term “experimental treatment”. However, good clinical governance structures need to be in place and patients need to be aware of the OL status of the therapy they are being offered.

There is incontrovertible evidence to support the risks of ULM in patient care. There are two strategies healthcare professionals can deploy to mitigate these risks.

The first is about giving patients the following information,

- Is the medicine licensed for the condition they are being treated for and is it being used in the correct dose and formulation?
- What is the evidence that a ULM is safe, effective and best for them?
- Is there a licensed alternative?
- What are the potential side effects and what steps will be taken to monitor and deal with them?

Prescribers need to be open with patients and always inform them when an ULM is being used; whether as a SM, OL, or TULM. Healthcare professionals have a responsibility to educate and inform patients and carers about the meaning of terms like unlicensed and off label. They should provide adequate information and assurances that includes a lay explanation of the evidence behind the choice of an ULM. Pharmacist must work collaboratively with prescribers to ensure patients’ needs are met and that only licensed medicines are used when a generic prescription is written and there is a choice between a licensed and unlicensed product.

The second strategy relates to the policies and procedures that are in place to protect patients from harm caused by ULM. This is a shared responsibility between professionals and their employing institutions; the professional bodies such as the Royal Colleges (or their equivalent elsewhere); the professional regulators such as the GMC in the UK; and the medicines regulators such as the MHRA and the EMA. All four need to look at their policies and guidelines and adapt them to modern issues around the increasing use of ULM despite new licensed medicines coming on to market.

### Governance considerations

Employing authorities need to have robust clinical governance policies and processes in place to ensure that where ULM are used there is an adequate evidence base, there is appropriate patient information available, that there is good record keeping and that there is consent. These policies must be readily available [For example, the East of England NHS [37]] and subjected to rigorous audit. The professional bodies and the professional regulators need to cooperate to ensure that guidance is consistent—preferably across the EU. The experience of the UK sees the GMC and Royal College of Paediatrics and Child Health guidelines differ in several areas [22, 38]. Professional bodies need to work with the medicine regulators to make changes to the licensing processes overcoming some of the issues. Stricter controls are needed to ensure that unlicensed “copycat” medicines are not used where a licensed product containing the same substance is available. Obtaining a marketing authorisation for any medicine is a long, expensive process, and the continued use of ULM jeopardises future ethical research and development. We believe that where common practice, with proper prospective audit, has shown a medicine to be safe and effective when used OL, the licence should be extended without the need for formal clinical trial evidence. We appreciate that the current regulatory framework does not allow for this to occur, so we advocate a change in regulations to facilitate this new way of working. There are many medicines, such as low molecular weight heparins, that are not licensed for use under a certain age yet are prescribed safely and effectively on a daily basis, very often as part of international consensus guidelines [39]. Extending the licence in these circumstances without having to go through a lengthy process would be advantageous to all concerned and would bring those products into structured pharmacovigilance procedures. There should also be a discussion between all concerned as to whether, or how, this principle could be extended to less commonly used medicines where ULM are part of evidence-based protocols utilising good, published, and audited evidence of safety and efficacy as described by Mellor and colleagues [20].

The use of tranexamic acid in the management of haemorrhage from major trauma is a good example. It is not licensed for this use, yet there have been reports of patient benefit when administered within 1 h of injury and it has been recommended by NICE [40, 41]. There is a need for legislators and regulators to examine how policies and procedures can be put in place to encourage appropriate innovation. Professional and advisory bodies could also help by coordinating and supporting efforts to produce and standardise robust clinical governance documentation and robust audit tools.

### Summary

The use of ULM is widespread and is likely to increase, especially in the field of less common or rare diseases. In order to ensure the safe and efficacious use of ULM, robust clinical governance processes need to be in place with adequate information available for patients. Healthcare providers need to review their use of ULM ensuring that licensed medicines are used wherever possible. Healthcare commissioners must be aware that the fiscal pressure they apply to healthcare providers may drive poor practice with respect to the use of ULM. Professional bodies and regulators need to review their guidelines and regulations ensuring that they are fit for purpose and can facilitate the use of ULM, especially OL use, where possible to ensure that innovation and advancing medical knowledge is not curtailed. Individual healthcare professionals need to be more aware of the needs and concerns of their patients and carers, must take steps to educate and inform them about ULM, and ensure that where there are concerns, these are addressed. More data is needed to help encourage these changes, and further research is important. Finally, TULM should not be used where a licensed alternative is available, especially where the active compound of both is identical.

This review has also identified important areas for future research.

- What is the true extent of ULM use and to what extent are ULM used where a licensed alternative is known?
- What is the influence of geographical or social factors on how healthcare providers interpret guidance? This will help us understand clinician motivations in this field.
- What governance structures are in place in various countries and how can they be strengthened?
- Can robust audit tools be developed to assist in monitoring this important area of clinical practice?
